# Circular Dichroism Spectra of α-Chymotrypsin–SDS Solutions Depend on the Procedure of Their Preparation

**DOI:** 10.1021/acsomega.2c02438

**Published:** 2022-06-28

**Authors:** Karolina Stachurska, Urszula Marcisz, Maciej Długosz, Jan M. Antosiewicz

**Affiliations:** Biophysics Division, Institute of Experimental Physics, Faculty of Physics, University of Warsaw, Pasteura 5 Strasse, 02-093 Warsaw, Poland

## Abstract

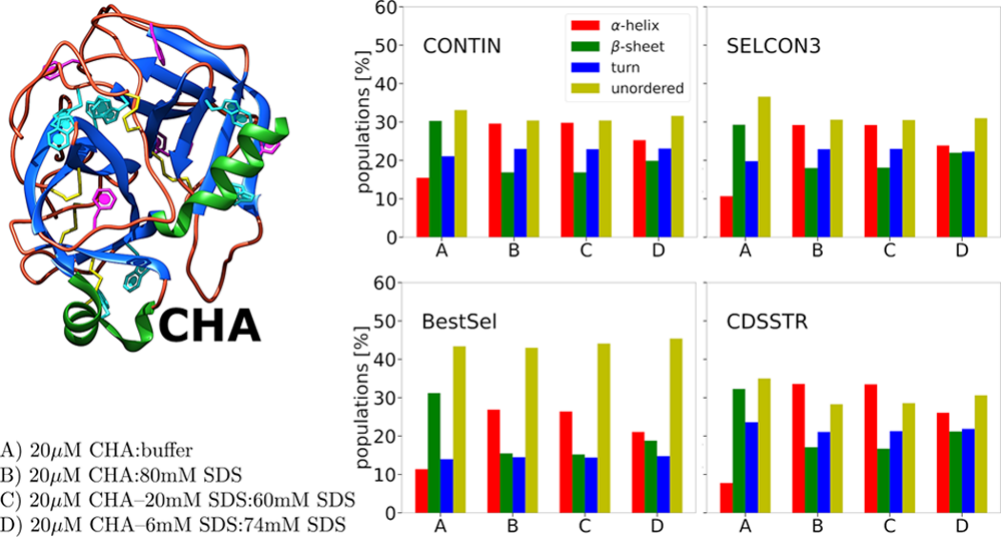

We recorded the far- and near-UV circular dichroism (CD) spectra of solutions of α-chymotrypsin and sodium dodecyl sulfate (SDS) with the final surfactant concentration significantly above the critical micellization concentration. Solutions were prepared using three different procedures. The reference procedure was to mix the chymotrypsin solution with the SDS solution once, immediately achieving the final SDS concentration. In alternative procedures, the protein solutions initially contained some SDS and were mixed with pure SDS solutions at a concentration to provide the same final surfactant as the reference mixing. We demonstrate that the supplementation to the selected final concentration of SDS of the pure chymotrypsin solution leads to different CD spectra than the supplementation to this final concentration of SDS in the chymotrypsin solution containing a small concentration of a few millimolar SDS. These differences disappear when the initial concentration of SDS in the protein solution, which we then supplement to the indicated final concentration, is higher. This suggests the irreversibility of the processes caused by the addition of SDS to chymotrypsin and the influence of the initial amount of this surfactant on the processes occurring with its further addition to the solution. For quantitative analysis of far-UV CD spectra in terms of populations of protein secondary structure elements, we used four well-established software packages. All programs consistently indicate that the observed differences in the far-UV CD spectra can be explained by the differences in the increase in the population of helical forms in chymotrypsin under the influence of SDS.

## Introduction

Research on protein–surfactant interactions has a long history owing to their importance in the pharmaceutical, food, and cosmetics industries.^1−9^ Sodium dodecyl sulfate (SDS), an anionic detergent known to unfold globular proteins, is most commonly used in these studies as a representative surfactant.^2,3,10,11^

The research described in this paper tackles problems related to the free protein fraction in the presence of SDS, reversibility of protein–SDS binding, and reversibility of changes induced by SDS in protein structures that thus far have not been fully explained.^3^

Otzen and coauthors assumed equilibrium of the free protein and an initial complex of the protein with a certain number of bound SDS molecules followed by an irreversible one-step structural change of the complex.^12−14^ However, in their more recent work on SDS-triggerd protein unfolding, they do not define this process as irreversible.^5,15,16^ Bhuyan,^17^ who investigated the action of SDS on the ferrocytochrome *c*, noted minima in the dependence of the observed rate constants for protein conformational transitions on the SDS concentration, which were interpreted as an indication that these transitions are reversible.

Others proposed that protein–surfactant solutions contain not only different types of protein–surfactant complexes but also free protein molecules, free surfactant molecules, and surfactant micelles (provided that the surfactant concentration is above the critical micelle concentration (CMC)).^4^

On the other hand, Takeda and coauthors^3,18^ stated that there is no free protein in a protein–SDS solution and that denaturation of proteins by surfactants is not easily reversible by any means. In addition, they pointed out discontinuous mobility changes of surfactant–protein complexes observed in capillary electrophoresis (CE) experiments with an increasing SDS concentration (first reported by Sasa and Takeda for bovine serum albumin (BSA)^19^). These changes are related to distinct protein–SDS complexes. According to Takeda and Moriyama,^3^ there are specific numbers of surfactant molecules that the protein can form complexes with. Therefore, there are certain threshold SDS concentration values corresponding to different numbers of surfactant molecules bound by each protein. When a protein solution is mixed with an SDS solution in which the concentration of the surfactant falls between two consecutive threshold values, each protein binds the same specific number of SDS molecules and subsequently assumes a certain conformation, in one irreversible step, without changing the amount of the bound surfactant.

Our contribution to the field of protein–surfactant interactions, presented in the current work, is evidence that far- and near-UV CD spectra of α-chymotrypsin (CHA)–surfactant solutions (and thus the secondary and tertiary structures^20^ of CHA) depend not only on the concentration of the surfactant but also on the procedure in which this concentration is established. In addition, we present CD spectra of CHA–SDS solutions below and near the critical micelle concentration whose properties can be related to the discontinuous mobility changes of the surfactant–protein complexes discussed above.

The research presented in the current paper originated from the following circumstances. We attempted to interpret the reaction progress curves recorded in the stopped-flow spectrometer after mixing chymotrypsin and SDS solutions. Numerical analysis of these curves did not allow us to conclude whether the occurring single-molecule transformations of the protein are reversible or unidirectional. Therefore, we conducted a series of experiments in which we mixed protein solutions with a certain amount of SDS already added with solutions of pure SDS. We thought that such experiments would allow us to answer the question about the reversibility of SDS-triggered conformational transitions of chymotrypsin. From these new experiments it became apparent that the CD spectra of chymotrypsin–SDS solutions depend on the procedure of their preparation.

## Materials and Methods

### Preparation of Solutions

All chemicals used in the present work were obtained from ROTH: Chymotrypsin (ROTH, ≥1000 USP-U/mg, for biochemistry, Art-Nr. 0238.3), sodium dodecyl sulfate (ROTH, ≥99%, for electrophoresis, biochemistry, and molecular biology, Art-Nr. 2326.1), sodium dihydrogen phosphate monohydrate (ROTH, ≥98%, p.a., ACS, Art.-Nr. K300.1), disodium hydrogen phosphate dehydrate (ROTH, ≥98%, p.a., ACS, Art.-Nr. 4984.2), sodium chloride (ROTH, ≥98%, p.a., ACS, ISO, Art.-Nr. 3957.1), and L-tryptophan (ROTH, ≥98,5%, Ph. Eur., for biochemistry, Art.-Nr. 4858.1). Reagent weights for buffers were made with an accuracy of 1 mg.

Chymotrypsin and SDS were dissolved in sodium phosphate buffer (10 mM, pH = 7), prepared using ultrapure Millipore Milli-Q water (resistance 18.2 MΩ cm^–1^). Solutions used in the experiments were prepared by diluting appropriate stock solutions of chymotrypsin and SDS. The concentration of the stock protein solution was ∼50 μM, controlled spectrophotometrically (with a UV-2401-PC Shimadzu spectrometer) using ϵ_282nm_ = 51 000 M^–1^ cm^–1^. The concentration of the stock SDS solution, 400 mM, was prepared by weight. SDS weights were prepared with an accuracy of 0.01 mg. Dilution and/or mixing of the stock solutions secures the best consistency of concentrations of the following parent solutions: 40 μM CHA, 20 μM CHA, 40 μM CHA with 6 mM SDS, 20 μM CHA with 6 mM SDS, 40 μM CHA with 20 mM SDS, 20 μM CHA with 20 mM SDS, 80 mM SDS, 74 mM SDS, 60 mM SDS, and 6 mM SDS. The above parent solutions were prepared from the stock solutions at least 1 h before the spectrophotometric experiments.

### Spectrophotometric Measurements

Circular dichroism spectra were collected using a Chirascan Plus (Applied Photophysics Ltd.) spectrometer that enables simultaneous measurement of CD and absorbance spectra. For the far-UV range (190–260 nm), a 1 mm cell was used. For the near-UV range (250–350 nm), a 10 mm cell was used. CD spectra of either CHA solutions or the buffer were scanned with a 0.5 s integration, 0.5 nm step resolution, and 1 nm bandwidth. Each spectrum is the average of 20 scans; thus, the total integration time for each point of the spectrum is 10 s. Prior to spectra measurements, the CD baseline was recorded with an empty cell holder and with a 10 s integration. From each recorded spectrum of CHA solution, the corresponding smoothed buffer spectrum was subtracted. Buffer spectra were smoothed using the Savitsky–Golay method^21^ implemented in an in-house software. All spectroscopic measurements were carried out at 20 ± 0.01 °C.

In the primary experiments, spectra were recorded for the solutions listed in Table 1. We used an initial concentration of CHA of 20 μM in the far-UV and 40 μM in the near-UV. The concentration of 10 μM protein (after 1:1 dilution) was necessary to record spectra in the far-UV range, beginning from 190 nm. On the other hand, the protein concentration of 20 μM (after 1:1 dilution) secures stronger signals in the near-UV region.

**Table 1:** List of Mixing of Parent Solutions in a 1:1 Volume Ratio To Obtain Solutions Tested in the Primary Part of This Work

component 1	component 2
40 μM CHA	buffer
40 μM CHA	80 mM SDS
40 μM CHA–6 mM SDS	74 mM SDS
40 μM CHA–20 mM SDS	60 mM SDS
20 μM CHA	buffer
20 μM CHA	80 mM SDS
20 μM CHA–6 mM SDS	74 mM SDS
20 μM CHA–20 mM SDS	60 mM SDS

After mixing a given pair of parent solutions, spectra were recorded, and afterward, the cuvette was cleaned and dried. The next pair of the parent solutions was then mixed, and spectra recording followed. The recording of 20 replicates of the spectrum plus the time spent washing and drying the cuvettes took about 40 min for the far-UV experiments and 50 min for the near-UV experiments. After the last pair of parent solutions was mixed, we repeated spectra recording for the first pair using the already prepared mixture. Thus, recording of the next spectrum for each pair commenced about 160 min for the far-UV or 200 min for the near-UV after preparation of a given mixture. Three cycles of the measurements were done for the far-UV range, and two cycles were done for the near-UV range. Therefore, the stability of the spectra of a given mixture means that no visible changes occurred within at least 320 min (far-UV) or at least 200 min (near-UV) after preparation (as pointed out above, the recording of 20 individual scans per spectrum takes 40–50 min).

In the additional experiments, the spectra were recorded for the solutions listed in Table 2. Spectra were recorded only once as averages of 20 scans with a 0.5 s integration time per point at each scan, 1 h after mixing the appropriate parent solutions. Recording of 20 scans per spectrum took 40 min for the far-UV range and 50 min for the near-UV range.

**Table 2:** List of Mixing Parental Solutions in a 1:1 Volume Ratio To Obtain Test Solutions in Additional Experiments

component 1	component 2
40 μM CHA	buffer
40 μM CHA	80 mM SDS
40 μM CHA	6 mM SDS
40 μM CHA–6 mM SDS	buffer
20 μM CHA	buffer
20 μM CHA	80 mM SDS
20 μM CHA	6 mM SDS
20 μM CHA–6 mM SDS	buffer

In 10 mM phosphate buffer, pH 7, and a temperature of 25 °C (5 °C higher than that used here), the CMC of SDS is 4.61 ± 0.01 mM.^22^ Thus, 6 mM SDS in some of the parent solutions described above is slightly more than the CMC. However, after mixing 6 mM SDS with a CHA solution in a 1:1 volume ratio or after mixing a CHA solution containing 6 mM SDS with the buffer, the surfactant concentration becomes lower than the CMC.

## Results and Discussion

### Far-UV Spectra

**Figure 1:**
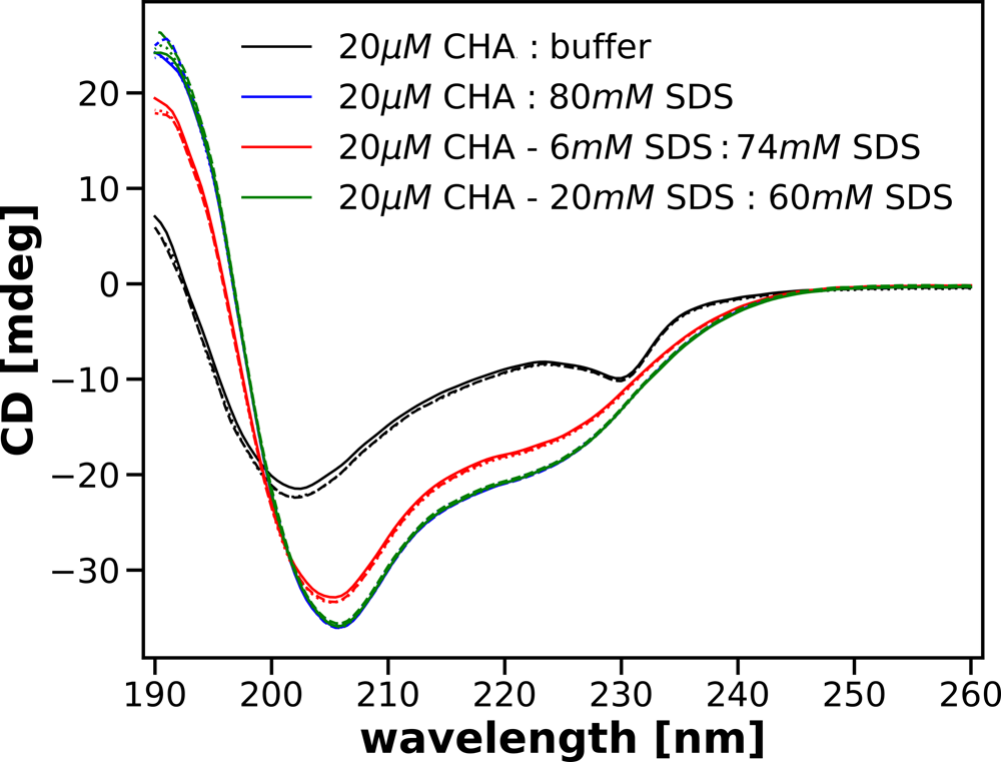
Far-UV CD spectra of 10 μM solutions of CHA obtained after mixing the parent solutions specified in the legend.

Figure 1 presents the far-UV CD spectra of solutions of 10 μM CHA in 10 mM phosphate buffer with and without 40 mM SDS. The spectra recorded just after mixing the appropriate parent solutions are shown as solid lines. Dashed lines correspond to spectra recorded 160 min after the first spectra. Dotted lines correspond to spectra recorded 320 min after the first spectra. As can be seen, the spectra are stable for at least 320 min.

**Figure 2:**
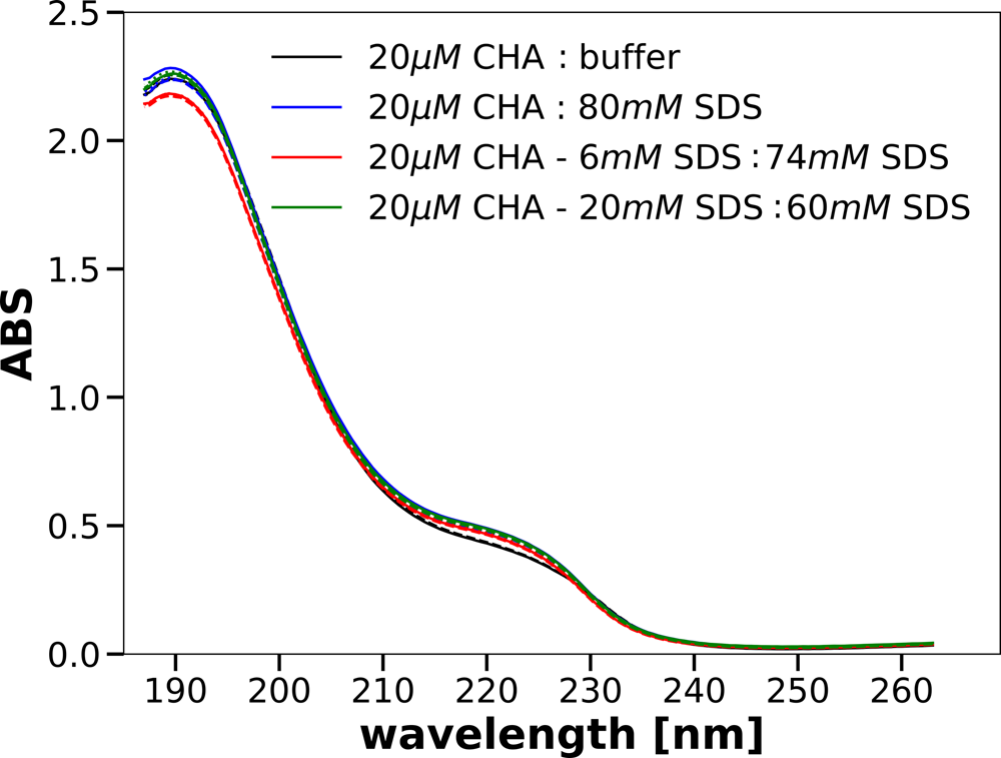
Far-UV absorption spectra of 10 μM solutions of CHA obtained after mixing the parent solutions specified in the legend.

The spectra recorded for solutions obtained after mixing a solution of 20 μM α-chymotrypsin with the buffer and a solution of 20 μM α-chymotrypsin with 80 mM SDS were used as reference spectra for our discussion. These spectra qualitatively agree with those reported in the literature.^23−26^ We compare the second spectrum with two spectra recorded for solutions where the final SDS concentration of 40 mM was reached in two steps. In the first case, a solution of 20 μM α-chymotrypsin containing the addition of 20 mM SDS was mixed with a solution of 60 mM SDS. In the second case, a solution of 20 μM α-chymotrypsin containing the addition of 6 mM SDS was mixed with a solution of 74 mM SDS. The spectrum of the solution obtained after mixing 20 μM protein containing the addition of 20 mM SDS with a solution of 60 mM SDS is identical with the spectrum of the solution obtained by mixing 20 μM chymotrypsin with 80 mM SDS. However, when 20 μM protein containing the addition of 6 mM SDS is mixed with with a solution of 74 mM SDS, the recorded spectrum is different. We will see similar effects in the case of the near-UV spectra, which will be presented later in this work, and these are the most important observations in our project that have not been described so far by other researchers. Figure S1 in the Supporting Information shows examples of several other mixing experiments that yielded similar sets of far-UV CD spectra. At the same time, as shown in Figure 2, the absorption spectra in the range 210–230 nm in the presence of 40 mM SDS almost overlap, no matter how this final concentration was obtained in solution, and are different from the native protein absorption spectrum.

We can also see that the spectral fragments close to 190 nm do not cluster as in the range 210–230 nm. However, the amount of light with wavelengths close to 190 nm reaching the detector is small, which significantly reduces the accuracy of the measurements. The absorption values shown in Figure 2 are values corrected for buffer absorption. The light absorption by the buffer around 190 nm is significant such that the total absorption of the solution at these wavelengths exceeds 3. Consequently, the absorption and circular dichroism spectra are not measured as accurately as for longer wavelengths. To improve the accuracy of recorded spectra below 200 nm, it would be necessary to use a shorter optical path. We did not do so, but we believe that it has no bearing on the conclusions drawn from the research described in this paper.

Data processing and deconvolution calculations to obtain secondary structure patterns from the CD spectra in the FUV range were performed using four software packages: BeStSel,^27,28^ SELCON3,^29^ CDSSTR,^30^ and CONTIN.^31,32^ The latter three programs are included in the CDPro software package.^33^

In the BeStSel program, five elements of the secondary protein structure are distinguished. These are helices, antiparallel β-sheets, parallel β-sheets, turns, and others. In the SELCON, CDSSTR, and CONTINUOUS programs, there are regular α-helices, distorted α-helices, regular β-sheets, distorted β-sheets, turns, and unordered. To simplify the comparative analysis, we reduced the number of distinguishable elements to four: α-helices, β-sheets, turns, and other (or unordered). The comparison is shown in Table 3, while the detailed results are shown in Tables S1–S4 in the Supporting Information. The Supporting Information also outlines the procedure for moving from the detailed results in Tables S1–S4 to the results shown in Table 3.

**Table 3:** Average Results of Analysis of Populations of Different Elements of the Secondary α-Chymotrypsin Structure in Four Mixing Experiments^a^

		secondary structure type			
EXP	program	α-helix	β-sheet	turn	unordered
A	SELCON3	10.7	29.3	19.8	36.6
	CDSSTR	7.8	32.3	23.6	35.0
	CONTIN	15.5	30.3	21.1	33.1
	BeStSel	11.4	31.2	14.0	43.4
B	SELCON3	29.2	18.0	22.9	30.6
	CDSSTR	33.6	17.1	21.1	28.3
	CONTIN	29.6	16.9	23.0	30.4
	BeStSel	26.9	15.5	14.5	43.0
C	SELCON3	29.2	18.1	23.0	30.5
	CDSSTR	33.5	16.7	21.3	28.6
	CONTIN	29.8	16.9	22.9	30.4
	BeStSel	26.4	15.2	14.4	44.1
D	SELCON3	23.9	22.0	22.3	31.0
	CDSSTR	26.1	21.2	21.9	30.6
	CONTIN	25.3	19.9	23.1	31.6
	BeStSel	21.1	18.8	14.8	45.4

a Conditions: 20 μM CHA with phosphate buffer (EXP A), 20 μM CHA with 80 mM SDS (EXP B), mixture 20 μM CHA–20 mM SDS with 60 mM SDS (EXP C), and mixture 20 μM CHA–6 mM SDS with 74 mM SDS (EXP D) obtained from four programs indicated.

Hunt and Jirgensons^24^ showed that in the presence of a saturating amount of dodecyl sulfate, CHA adopts a conformation with a higher helix content than the native protein. The populations of the secondary structure elements, presented in Table 3, are in line with these findings. Moreover, Table 3 shows something new that seems interesting and, to our knowledge, has not been described so far, namely, all of the employed programs consistently show that the increase in the α-helical population in the presence of 40 mM SDS depends on the manner in which the concentration of the surfactant is brought to this value. This observation is the most important result documented in our work.

When a 20 μM solution of CHA containing the addition of 20 mM SDS is mixed with a solution containing 60 mM SDS, the populations of the α-helices and β-sheets are the same as those observed when a 20 μM solution of CHA is mixed with a solution of 80 mM SDS. The largest difference between the pairs of compared values is 0.5%. However, when a 20 μM solution of CHA containing the addition of 6 mM SDS is mixed with a solution of 74 mM SDS, the populations of α-helices are smaller by 4.3–7.5% and β-sheets are larger by 3.0–4.5% than in the two previous cases, although the final concentration of the surfactant is the same. All four programs give similar results in terms of differences in the populations of the helices and sheets. However, there are differences between the BeStSell program and the other three programs regarding changes in the populations of twists and unstructured parts of the polypetide chain. Nevertheless, it seems valid to conclude that CHA adopts different secondary structures when the final 40 mM concentration of SDS is determined in the two steps described above.

### Near-UV Spectra

The results obtained in the analysis of the secondary structure of CHA naturally induce analogous research on the tertiary structure of this protein to be carried out. For this purpose, we conducted similar tests to those described above in the near-UV range.

**Figure 3:**
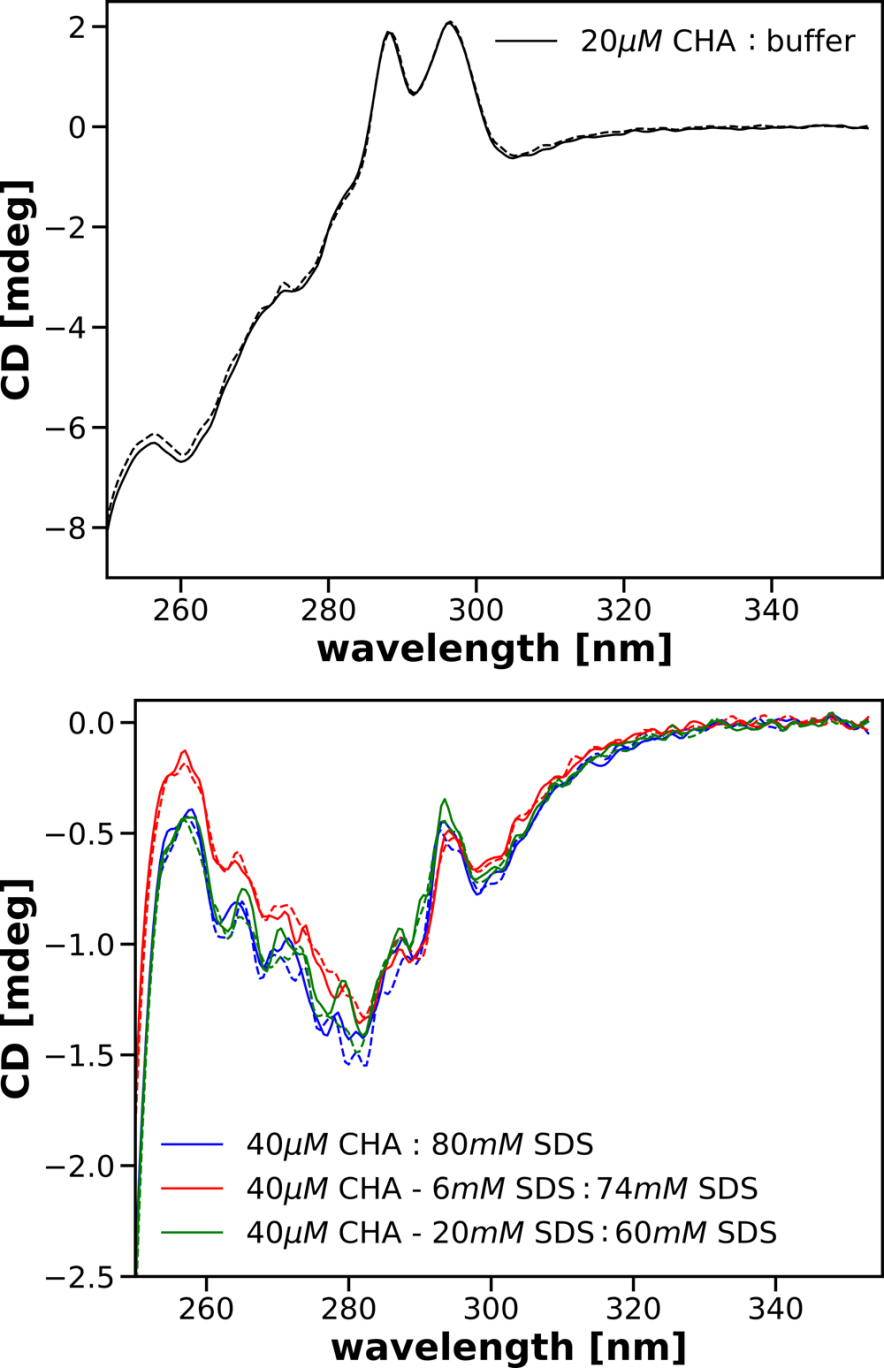
Near-UV CD spectra of 20 μM solutions of CHA obtained after mixing the parent solutions specified in the legends.

**Figure 4:**
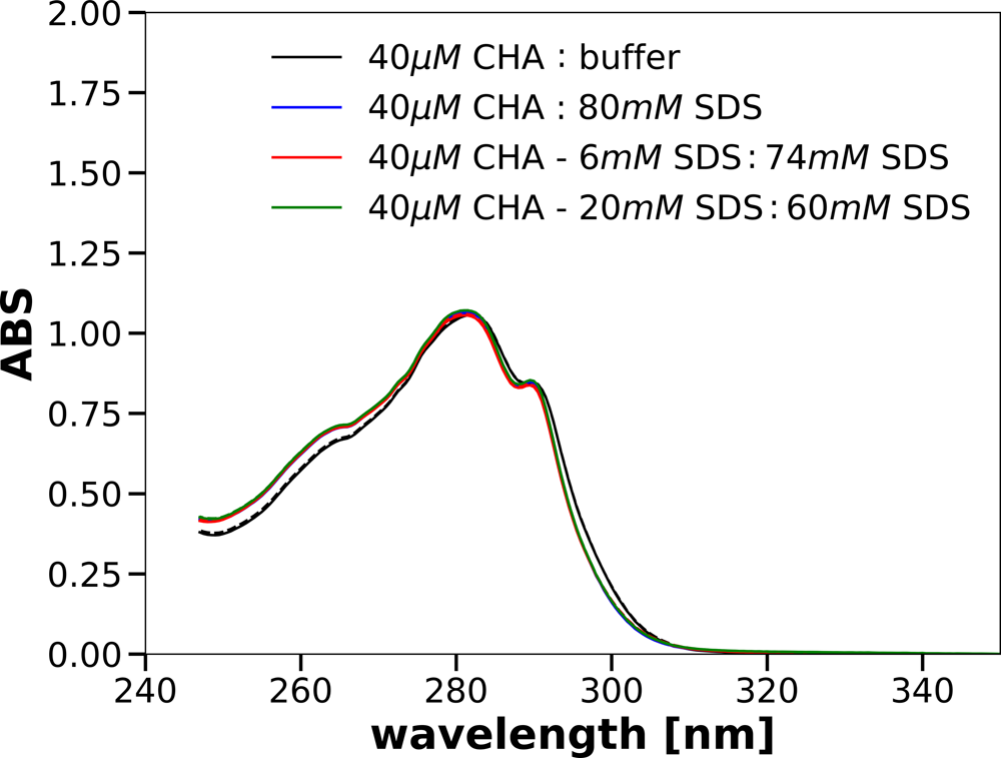
Near-UV absorption spectra of 20 μM solutions of CHA obtained after mixing the parent solutions specified in the legend.

Figure 3 shows spectra recorded in the near-UV range for 20 μM CHA solutions without addition and with the addition of 40 mM SDS. The solid lines show the spectra recorded immediately after preparing the solutions. The spectra shown by dashed lines were recorded 200 min after the spectra represented by the corresponding solid lines. Similarly, as in the far-UV case, the CD spectra obtained without SDS and with its 40 mM concentration qualitatively agree with those presented in the literature.^24^ The most interesting thing in the spectra shown in Figure 3 are the differences in the range of 250–280 nm between the spectra of the solution obtained by mixing a 40 μM CHA solution with the addition of 6 mM SDS with a 74 mM SDS solution and the spectra of the other two solutions containing a final concentration of 40 mM SDS. In this spectral range, the CD bands arise from the aromatic amino acid phenylalanines and tyrosines as well as from disulfide bonds.^20,24^ In the 280–350 nm range, the spectra of the solutions containing 40 mM SDS are the same no matter how this final SDS concentration was reached. We conclude that the tertiary structure of CHA in the vicinity of tryptophan residues does not depend on the procedure of determining the final 40 mM concentration of this surfactant. Figure 4 shows the absorption spectra recorded in the near-UV range. As can be seen, the absorption spectra of the solutions containing 40 mM SDS are the same no matter how this concentration was achieved. On the other hand, the absorption spectrum of a solution without SDS is clearly different. These observations are analogous to those made for the far-UV absorption spectra. Moreover, the absence of absorption at 350 nm leads to the conclusion that there is no aggregation of CHA in our solutions.^34,35^

Another interesting observation is that the spectra recorded for solutions with the final concentration of SDS of 40 mM, obtained in two steps (40 μM CHA with 6 mM SDS:74 mM SDS and 40 μM CHA with 20 mM SDS:60 mM SDS), recorded just after the mixing of the appropriate parent solutions, almost perfectly overlap with the spectra recorded 200 min later (Figure 3). The reproducibility of the spectra is the same as in the case of 40 μM CHA:80 mM SDS or diluting the protein solution with the buffer, 40 μM CHA:buffer. This indicates that the tertiary structures of α-chymotrypsin in these final solutions are stable and that the small difference between the spectra visible in the range 250–270 nm in the bottom panel of Figure 3 is meaningful.

We can therefore conclude that not only the secondary structure but also the tertiary structure of CHA in a solution containing 40 mM SDS may depend on the procedure used to achieve this fnal concentration of surfactant. Since the differences in the CD spectra are visible around 260 nm, we can assume that the structural differences are present in the vicinity of phenylalanine residues and possibly tyrosines and disulfide bridges. In the spectral regions above 280 nm, where the CD signals arise due to tryptophans, the spectra of CHA in the presence of 40 mM SDS obtained by different mixings are the same.

The results shown in Figures 1 and 3 are well reproducible in independent experiments with new preparations of the stock solutions. Thus, the secondary and tertiary structures of CHA in the presence of the micellar concentration of SDS are determined not only by the value of this concentration but also by the course of the mixing process.

### Possible Significance of CHA Interactions with SDS Monomers

The observation of the differences discussed above requires that the concentration of SDS added to the CHA solution in the first step is relatively small. In our experiments, we used a SDS concentration of 6 mM, which is a value slightly exceeding the CMC for SDS, amounting to 4.61 ± 0.01 mM in our experimental conditions.^22^ Mixing a parent solution, containing 6 mM SDS, with a solution not containing the surfactant, done in the experiments of the second type (see Table 2), results in a solution with 3 mM SDS, thus below the CMC. Besides having SDS concentrations either above or below the CMC, these parent solutions and their mixing should be analyzed in terms of the concentration of SDS micelles. On the basis of the literature data, at experimental conditions similar to those of our experiments,^36,37^ the mean aggregation number of SDS micelles is on the order of 70, resulting in a concentration of micelles of roughly 20 μM. Thus, it appears that CHA interactions with single SDS molecules can be expected to play a significant role in solutions containing 6 mM surfactant.

**Figure 5:**
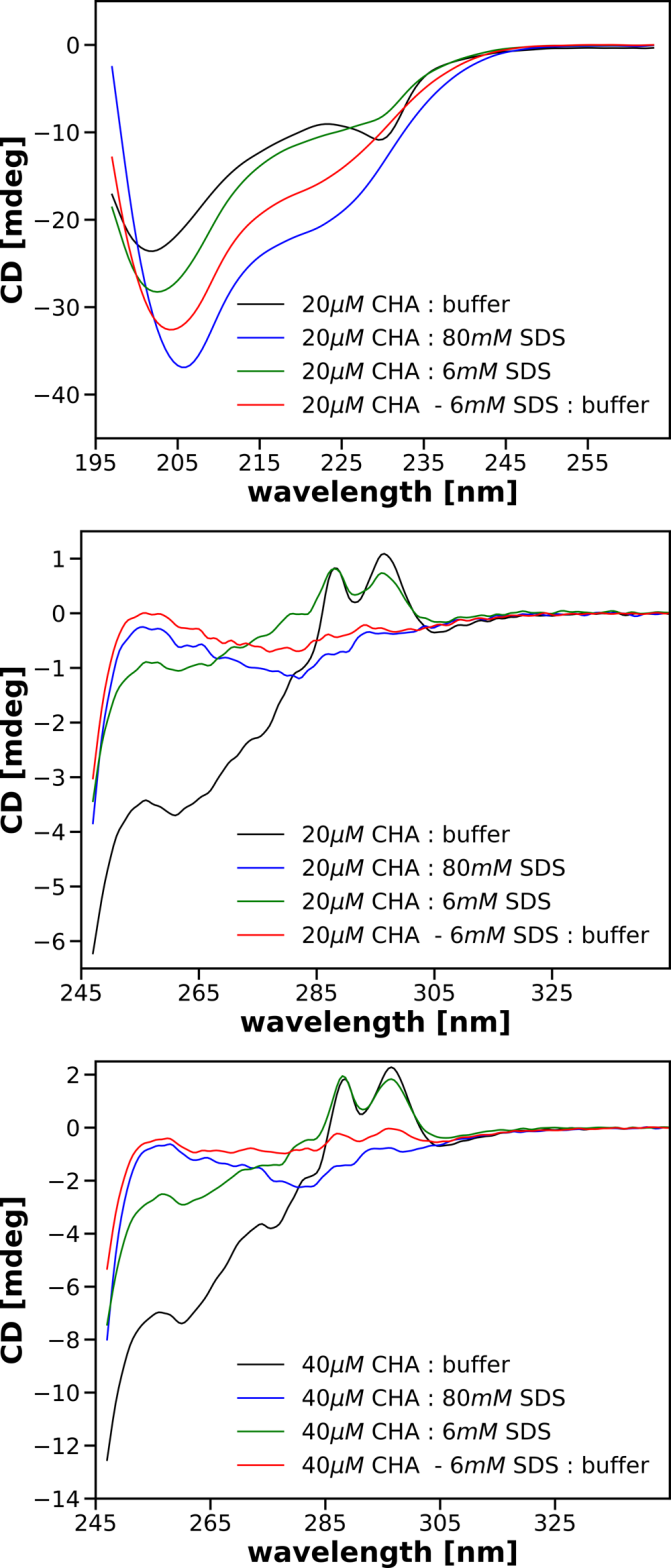
CD spectra in the far- (top) and near-UV (middle and bottom) recorded for solutions obtained after mixing the parent solutions specified in the legends. Spectra were recorded 1 h after mixing the parent solutions.

If the structural changes in a protein are caused by surfactant concentrations both below and above the CMC, as noted by Takeda and Moriyama,^38^ this indicates that the protein binds both individual surfactant molecules and micelles. The results presented in Figure 5 show that concentrations of SDS that are below or near the CMC (3–6 mM) induce changes in the secondary and tertiary structure of CHA. These changes are different than the changes induced in the presence of 40 mM SDS. Simultaneously, from the near-UV results, we can see that the spectra recorded for solutions having the same concentration ratio of CHA to SDS differ when the final concentrations are obtained using different mixing procedures. Note that the spectra were recorded 1 h after preparation of the solutions. Therefore, it can be expected that the molecular structures present in the solution are stable over time. This points to the irreversibility of binding monomers of SDS by CHA.

The observed differences in the spectra may seem surprising. For the solutions whose spectra are shown in the middle and bottom of Figure 5, the final SDS concentrations are the same and the ratios of CHA to SDS concentrations did not change when these solutions were prepared. Moreover, in the preparation of these solutions, the concentration of SDS was below or only slightly above the CMC. Thus, in these solutions mainly interactions of CHA molecules with single SDS molecules take place. It can be seen, however, that the observed differences in the spectra seem to be consistent with the capillary electrophoresis patterns of SDS–BSA complexes at representative SDS concentrations observed by Takeda and co-workers,^3,19^ provided that similar discontinuities in protein–SDS complex formations occur also in the case of CHA. In Figure 10 of Takeda and Moriyama,^3^ we can see that at 3 mM SDS, BSA exists in exclusively one form of a complex with the surfactant, whereas at 6 mM SDS, there is a small contribution of this form but another form of the complex is dominant. If the situation with CHA is similar, the observed differences in the spectra should be expected. This indicates that the structure of CHA (with a concentration of 20 or 40 μM, respectively) in the solution containing 6 mM SDS is different than the structure of the protein (with a concentration of 10 or 20 μM, correspondingly) in the solution containing 3 mM SDS. In addition, because the structure is stable, 2-fold dilution of the first solution, 20(40) μM chymotrypsin with 6 mM SDS, does not lead to the structure adopted by the protein when the corresponding concentration of the protein is mixed in a 1:1 volume ratio with 6 mM SDS.

**Figure 6:**
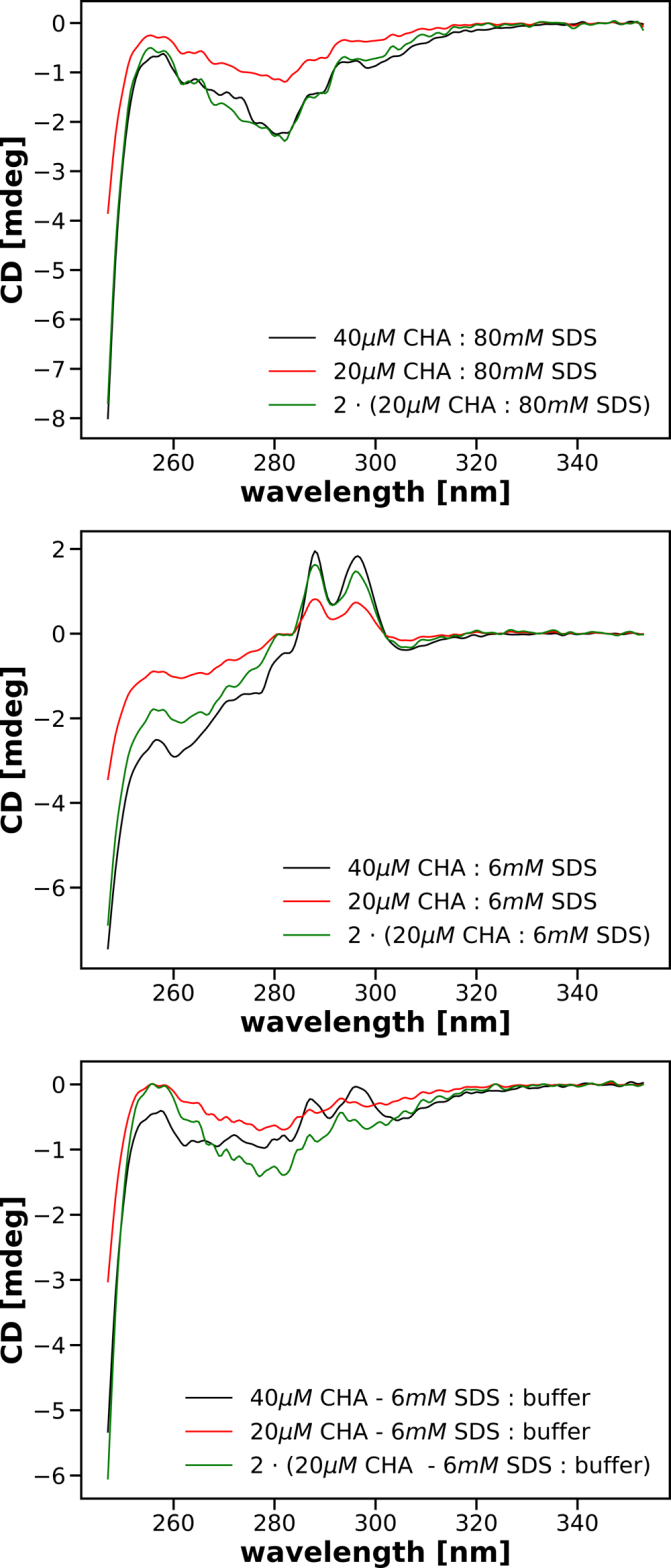
Comparison of near-UV CD recorded for solutions having a final concentration of 20 μM CHA with those having a final concentration of CHA equal 10 μM in the presence of the same concentration of SDS, either 40 or 3 mM. Details of mixings are shown in the corresponding legends. Spectra for 10 μM CHA were multiplied by a factor of 2 and are shown in green.

In Figure 6 we compare spectra obtained for final concentrations of CHA 10 and 20 μM in the presence of either 3 or 40 mM of SDS. In addition, we show spectra obtained from the spectra recorded for a final concentration of 10 μM CHA multiplied by a factor of 2. As can be seen, multiplication of the spectrum recorded after mixing 20 μM CHA with 80 mM SDS by 2 results in the spectrum almost perfectly fitting that recorded after mixing 40 μM CHA with 80 mM SDS. Visible differences most likely reflect the statistical noise accompanying the recording of spectra. The overlap between the spectrum recorded at 20 μM CHA concentration and the factor 2-multiplied spectrum recorded at 10 μM protein concentration is probably due to the 40 mM SDS concentration being the saturating concentration for both protein concentrations.

A substantially different picture emerges for spectra recorded for submicellar and near-critical concentrations of SDS. As can be seen in the central panel of Figure 6, the spectrum obtained after mixing 20 μM CHA with 6 mM SDS when multiplied by a factor of 2 superimposes quite well in the range 280–350 nm on the spectrum obtained after mixing 40 μM CHA with 6 mM SDS while being different for the remaining wavelengths.

On the other hand, in the bottom panel of Figure 6, we compare the spectrum of the solution obtained after mixing 20 μM CHA containing the addition of 6 mM SDS with the buffer multiplied by a factor of 2 and the spectrum of the solution obtained after mixing 40 μM CHA containing the addition of 6 mM SDS with the buffer. These spectra only match for wavelengths above 300 nm.

The observations described above seem consistent with the views of Takeda and Moriyama^3^ that there are no free protein molecules in solutions containing proteins and SDS regardless of the surfactant concentration and that the protein binding processes for SDS are virtually irreversible.

## Conclusions

The preparation of protein–surfactant solutions using the two-step procedure described in our work has not been previously considered. As we have shown using the example of CHA and SDS, such an approach is able to provide new insights into protein–surfactant interactions and the mechanism of surfactant-triggered structural transitions in proteins. We hope that our work will encourage others to perform similar studies on different systems using the vast arsenal of experimental methods that are available for investigations of surfactant–protein interactions.^5,15,39−41^ An interesting case study would be ferrocytochrome *c* for which SDS-induced conformational transitions appear to be reversible.^17^

## Supporting Information Available

Detailed results of the computational analysis of secondary structure patterns in α-chymotrypsin; additional examples of far-UV CD spectra, analogous to the spectra shown in Figure 1 (PDF)
