# Supplementary material for: Circular Dichroism Spectra of α-Chymotrypsin–SDS Solutions Depend on the Procedure of Their Preparation
Source: ACS Omega. 2022 Jun 28;7(27):23782–9. doi: 10.1021/acsomega.2c02438 (PMC9280762; doi:10.1021/acsomega.2c02438)
Supplement: Supplementary file 1 — ao2c02438_si_001.pdf [file ao2c02438_si_001.pdf]

Supporting information for: Circular dichroism spectra of α-chymotrypsin-SDS solutions depend on the procedure of their preparation.

Karolina Stachurska, Urszula Marcisz, Maciej Długosz, and Jan M. Antosiewicz*

Biophysics Division, Institute of Experimental Physics, Faculty of Physics, University of Warsaw, Pasteura 5 St., 02-093 Warsaw, Poland

E-mail: jantosi@fuw.edu.pl

Detailed results of the secondary structure population analysis in α-chymotrypsin in phosphate buffer without the addition of SDS and with 40 mM SDS obtained in one and two steps.

Processing and deconvolution calculations to obtain secondary structure patterns of α-chymotrypsin in phosphate buffer with and without addition of SDS were performed using four software packages: the BeStSel,[1,2] the SELCON3,[3] the CDSSTR,[4] and the CONTIN.[5,6] The latter three programs were used as part of the CDPro software package.[7] The results of these analyzes are presented in Tables S1 to S4. Our spectra were recorded in the 190-260 nm range. The BeStSel program analyzed our spectra in the 190-250 nm range, while in the case of the other three programs, it was in the 190-240 nm range.

The programs used distinguish different numbers of secondary structure elements in proteins. The BestSel distinguishes five elements in the secondary structure of proteins: helices, anti-parallel β-sheets, parallel β-sheets, turns and others. The other three programs distinguish six secondary structure elements in proteins: regular α-helices, distorted α-helices, regular β-strands, distorted β-strands, turns and disordered chains.

Looking at the results presented in Tables S1 to S4, we can see that the populations of secondary structures obtained from individual programs are stable in time and repeatable in both measurement series. Out of 80 groups of equivalent 6 results, only in three cases we observe single values significantly different from the other five. All these cases relate to the use of the BeStSel program. Thus, we believe that it is justified to consider the mean values obtained from the six numbers for each combination of the element of the secondary structure and the conditions for carrying out the measurements. These average values, also shown in Tables S1– S4, are collected in Table S5 for a more convenient comparison.

According to the authors of the CDPro software package,[7] if the results from three programs, SELCON3,[3] CDSSTR,[4] and CONTIN,[5,6] are similar then the analysis is reliable, and one can average the secondary structure fractions. Contrary, if the results from the three

programs differ considerably, then they are unreliable. There may be a problem with the Input data or the Input CD spectrum may not be well-represented in the reference proteins. As we can see from Table S5, within each of the experiments A through D, the results obtained from the three programs are quite similar. We can see only two inconsistencies in the results obtained from these three programs: clearly smaller population of the regular α-helix in the native α-chymotrypsin obtained from CDSSTR (2.6% vs. 6.0 and 6.3%) and clearly larger population of the distorted α-helix in the native α-chymotrypsin obtained from CONTIN (9.2% vs. 4.7 and 5.2%). The remaining results are pretty consistent. This assures us that the CD spectra obtained are of quite good quality. The results obtained from application of the BeStSel program (Table S5) are not very much different from those obtained with the CDPro programs suite. We can therefore trust the values of the alpha-chymotrypsin secondary structure population under different solution conditions obtained from these four programs.

The way the results are presented in Table S5 maintains the differences in the numbers of secondary structures analyzed in each program. In the CONTIN, SELCOM and CDSSTR programs we have regular α-helices (H_{α}^{R}), distorted α-helices (H_{α}^{D}), which in the BeStSel program are just helices. In turn, all programs recognize two types of sheets, denoted in Table S5 with the symbols S_{β}^{R} and S_{β}^{D}, but these types are named differently in the BeStSel program than in the other three programs. We also have minor differences in the nomenclature of the other two elements of the secondary structure. For these reasons, our last step was to reduce the recognizable elements of the structure to four. The final results are presented in Table 3 in the main text.

Additional examples of far UV CD spectra showing the differences observed in Figure 1 in the main article.

The observation that the spectrum obtained after mixing a chymotrypsin solution containing a small amount of SDS with a SDS solution such that the final surfactant concentration is

40 mM is not limited to 6 mM in the protein solution. Figure S1 shows such effects for SDS concentrations in the range of 2-16 mM. For the spectra shown in this figure, the initial protein concentration was 40 μM. At this concentration, the amount of UV radiation reaching the detector became too small to ensure an accurate measurement of the CD spectrum when the wavelength of the light fell below 205 nm.

The spectra shown in Figure S1 were recorded once, 5 minutes after mixing the appropriate stock solutions. Each part of Figure S1 shows the far UV CD spectrum recorded after mixing a 40 μM protein solution containing additionally a low concentration of SDS, denoted as 40 μM CHA - x mM SDS, with a (80$-$x) mM SDS solution. Therefore, the final SDS concentration after the mixing is 40 mM. Each of the obtained spectra is separately compared with two spectra. The first is the spectrum recorded after mixing 40μM protein solution with the buffer. The second spectrum, on the other hand, is the one recorded after mixing a 40 μM protein solution with an 80 mM SDS solution.

Figure S1 confirms that the spectra recorded after mixing a 40 μM chymotrypsin solution containing small amounts of SDS with a SDS solution at a concentration such that its concentration in the final solution was 40 mM, are different from the spectrum obtained after mixing a 40 μM protein solution with 80 mM SDS solution, despite the same final protein and surfactant concentration.

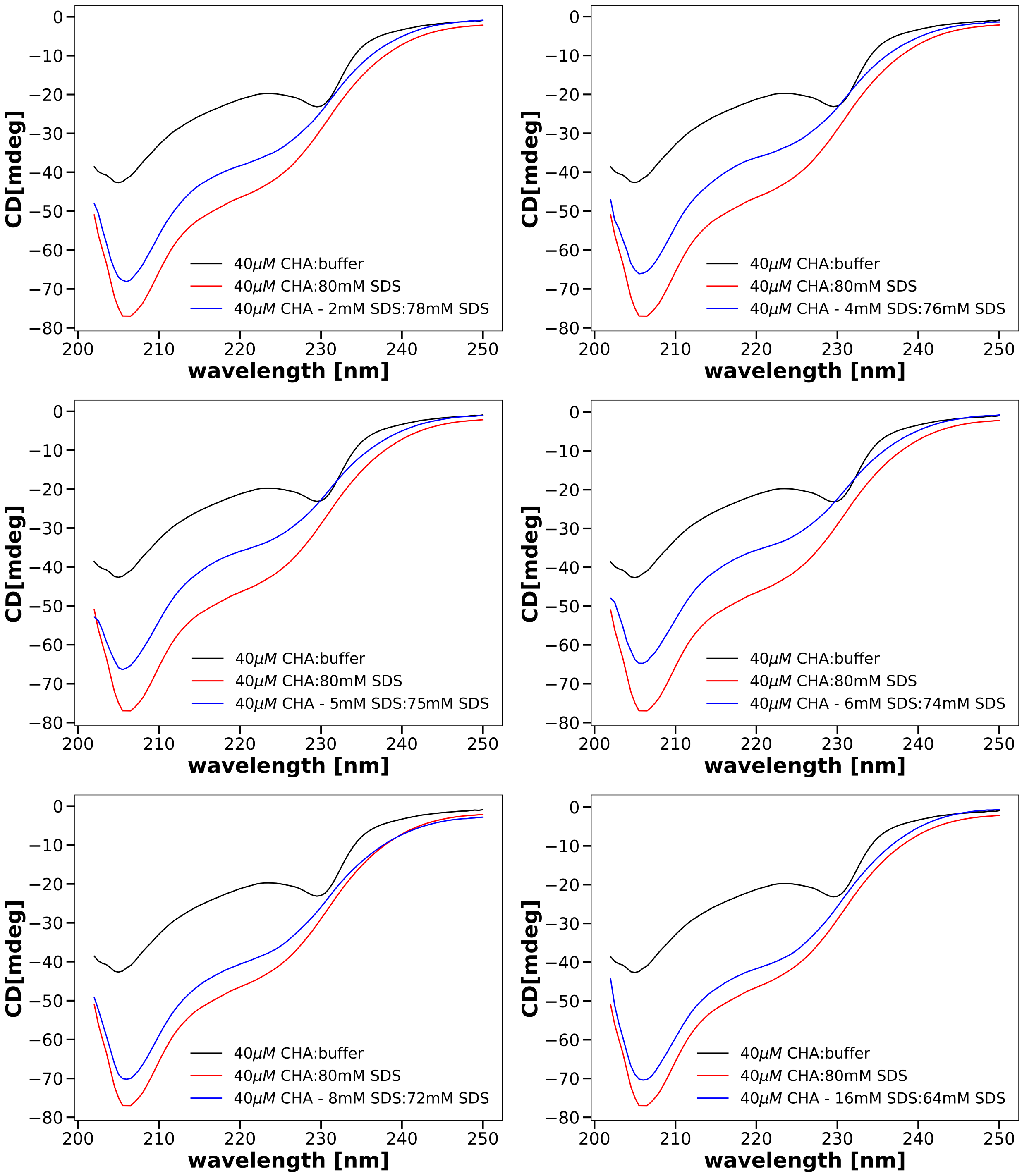

Figure S1: Circular dichroism spectra in the far ultraviolet region obtained after mixing CHA40μM:buffer (black), CHA40μM:SDS80mM (red), and CHA40μM - SDSxmM:SDS(80-x)mM (blue), registered after mixing.

Table S1: Results of the analysis with the BeStSel program,[1,2] of the spectra shown in Figure 1, and (separated with "/") of the spectra registered in an independent experiment. The three rows for each secondary structure element refer to three spectra registered for a given mixture just after its preparation, 160 min later and 320 min later.

	Percentage population of a secondary structure element			
Element	20μM CHA buffer	20μM CHA 80 mM SDS	20μM CHA-20 mM SDS 60 mM SDS	20μM CHA-6 mM SDS 74 mM SDS
Helix	10.9/11.6	27.2/28.0	26.9/27.1	17.1/22.2
	11.3/11.8	26.1/27.5	25.9/26.1	22.2/22.8
	11.5/11.5	26.4/26.3	26.1/26.2	22.4/20.1
**averages →**	**11.4**	**26.9**	**26.4**	**21.1**
Anti-parallel β-sheet	25.7/24.0	11.7/11.3	11.9/11.1	22.8/13.9
	23.9/22.7	13.0/11.6	13.5/11.9	14.7/13.8
	23.7/23.8	12.3/11.8	12.1/12.1	14.4/16.1
**averages →**	**24.0**	**12.0**	**12.1**	**16.0**
Parallel β-sheet	5.8/7.0	3.1/3.9	3.5/4.2	0.0/4.0
	7.5/8.2	3.7/4.0	1.9/2.3	3.2/4.1
	7.5/7.0	3.2/3.3	3.7/2.9	2.7/2.6
**averages →**	**7.2**	**3.5**	**3.1**	**2.8**
Turn	14.1/14.0	14.5/14.6	14.5/14.4	15.0/14.9
	13.9/14.0	14.1/14.8	14.0/14.5	14.4/15.3
	14.0/14.1	14.4/14.8	14.3/14.4	14.5/14.5
**averages →**	**14.0**	**14.5**	**14.4**	**14.8**
Others	43.5/43.3	43.5/42.2	43.3/43.2	45.1/45.1
	43.3/43.3	43.1/42.0	44.7/45.2	45.7/44.1
	43.4/43.6	43.6/43.8	43.8/44.4	45.9/46.6
**averages →**	**43.4**	**43.0**	**44.1**	**45.4**

Table S2: Results of the analysis using the program SELCON3,[3] of the spectra shown in Figure 1, and (separated with "/") of the spectra registered in an independent experiment. The three rows for each secondary structure element refer to three spectra registered for a given mixture just after its preparation, 160 min later and 320 min later.

	Percentage population of a secondary structure element			
Element	20μM CHA buffer	20μM CHA 80 mM SDS	20μM CHA-20 mM SDS 60 mM SDS	20μM CHA-6 mM SDS 74 mM SDS
regular α-helix	5.7/6.0	14.1/13.9	14.1/14.0	11.6/10.6
	6.0/6.0	14.2/13.7	14.0/14.0	12.0/10.6
	6.0/6.1	14.0/13.9	14.1/13.9	12.0/10.6
**averages →**	**6.0**	**14.0**	**14.0**	**11.2**
distorted α-helix	4.3/4.2	15.3/15.4	15.3/15.4	12.8/12.4
	4.6/5.2	15.2/15.2	14.8/15.0	13.4/12.6
	5.2/4.6	15.0/14.9	15.1/15.0	13.4/11.8
**averages →**	**4.7**	**15.2**	**15.2.**	**12.7.**
regular β-strand	18.4/17.5	10.3/10.6	10.3/10.4	13.4/13.9
	18.4/19.4	10.3/10.6	11.0/11.0	12.8/13.3
	18.9/18.6	10.6/11.0	10.4/10.8	13.2/14.9
**averages →**	**18.5**	**10.6**	**10.7**	**13.6**
distorted β-strand	10.6/10.3	7.2/7.6	7.2/7.5	8.3/8.7
	10.6/11.5	7.4/7.5	7.5/7.5	8.0/8.6
	11.0/10.7	7.3/7.4	7.4/7.5	8.0/8.8
**averages →**	**10.8**	**7.4**	**7.4**	**8.4**
turns	19.5/19.8	22.7/23.1	22.7/22.8	22.0/22.4
	19.5/20.9	22.7/22.9	23.3/23.4	22.7/22.5
	19.6/19.7	22.8/23.1	22.8/23.1	22.5/21.5
**averages →**	**19.8**	**22.9**	**23.0**	**22.3**
unordered	36.1/35.7	30.3/30.8	30.3/30.5	30.5/31.8
	36.1/39.0	30.6/30.6	30.5/30.6	29.9/32.0
	36.5/36.3	30.6/30.6	30.6/30.6	30.0/32.0
**averages →**	**36.6**	**30.6**	**30.5**	**31.0**

Table S3: Results of the analysis using the program CDSSTR,[4] of the spectra shown in Figure 1, and (separated with "/") of the spectra registered in an independent experiment. The three rows for each secondary structure element refer to three spectra registered for a given mixture just after its preparation, 160 min later and 320 min later.

	Percentage population of a secondary structure element			
Element	20μM CHA buffer	20μM CHA 80 mM SDS	20μM CHA-20 mM SDS 60 mM SDS	20μM CHA-6 mM SDS 74 mM SDS
regular α-helix	1.1/3.4	17.5/17.1	17.3/16.7	12.4/11.6
	1.9/2.8	17.5/17.2	17.3/17.2	15.3/11.3
	3.4/2.9	17.0/17.1	17.2/17.2	15.0/11.2
**averages →**	**2.6**	**17.3**	**17.2**	**12.8**
distorted α-helix	5.8/4.6	16.3/17.0	15.9/16.7	12.4/12.4
	5.2/5.4	15.6/16.9	15.6/16.3	15.0/13.2
	5.3/5.1	16.1/16.0	16.0/16.4	14.7/12.1
**averages →**	**5.2**	**16.3**	**16.3**	**13.3**
regular β-strand	20.9/19.8	10.0/9.0	9.9/8.9	13.5/13.8
	20.3/19.9	10.2/9.8	10.4/9.1	10.9/12.6
	20.3/20.4	9.6/10.1	9.5/8.8	11.1/13.8
**averages →**	**20.3**	**9.8**	**9.4**	**12.6**
distorted β-strand	12.4/11.9	7.4/6.9	7.5/7.2	8.8/8.7
	12.5/11.7	7.3/7.3	7.6/7.1	8.3/8.6
	11.6/11.7	7.3/7.4	7.4/7.1	8.2/9.1
**averages →**	**12.0**	**7.3**	**7.3**	**8.6**
turns	24.2/23.9	21.3/21.6	21.2/21.9	21.4/21.8
	23.6/23.6	20.7/21.0	20.3/21.5	21.6/22.8
	22.7/23.8	21.2/20.8	21.2/21.6	21.7/22.3
**averages →**	**23.6**	**21.1**	**21.3.**	**21.9**
unordered	34.0/35.0	27.5/28.7	28.7/28.6	31.2/31.2
	35.3/35.3	28.3/27.9	28.3/28.6	28.8/31.3
	35.2/35.5	28.6/28.8	28.5/29.0	29.8/31.5
**averages →**	**35.0**	**28.3**	**28.6**	**30.6**

Table S4: Results of the analysis using the program CONTIN,[5,6] of the spectra shown in Figure 1, and (separated with "/") of the spectra registered in an independent experiment. The three rows for each secondary structure element refer to three spectra registered for a given mixture just after its preparation, 160 min later and 320 min later.

	Percentage population of a secondary structure element			
Element	20μM CHA buffer	20μM CHA 80 mM SDS	20μM CHA-20 mM SDS 60 mM SDS	20μM CHA-6 mM SDS 74 mM SDS
regular α-helix	6.6/6.3	14.0/14.1	14.1/14.1	12.0/11.1
	6.3/6.3	14.4/13.8	14.4/14.0	12.0/11.1
	6.3/6.2	14.1/13.8	14.3/14.0	12.0/11.0
**averages →**	**6.3**	**14.0**	**14.2**	**11.5**
distorted α-helix	9.3/9.1	15.5/16.0	15.6/15.9	14.0/13.6
	9.1/9.8	15.8/15.7	15.5/15.5	14.1/13.6
	9.0/9.1	15.5/15.3	15.7/15.5	14.1/13.2
**averages →**	**9.2.**	**15.6**	**15.6**	**13.8**
regular β-strand	20.3/19.3	9.5/8.9	9.5/8.9	11.3/11.6
	19.6/18.3	9.3/9.3	9.6/9.6	11.2/11.3
	19.8/19.5	9.5/9.8	9.3/9.5	11.2/12.3
**averages →**	**19.5**	**9.4**	**9.4**	**11.5**
distorted β-strand	11.1/10.7	7.4/7.5	7.4/7.6	8.3/8.5
	10.9/10.4	7.5/7.6	7.4/7.5	8.3/8.5
	11.0/10.8	7.5/7.6	7.5/7.5	8.3/8.7
**averages →**	**10.8**	**7.5**	**7.5**	**8.4**
turns	20.6/21.2	23.1/23.2	22.9/23.2	22.9/23.4
	21.1/21.6	22.6/23.1	22.6/23.0	22.9/23.4
	20.9/21.2	22.9/23.0	22.9/23.0	23.1/22.9
**averages →**	**21.1**	**23.0**	**22.9**	**23.1**
unordered	32.1/33.5	30.5/30.4	30.5/30.3	31.4/31.9
	33.1/33.6	30.4/30.4	30.6/30.4	31.4/32.0
	33.0/33.2	30.5/30.4	30.4/30.3	31.2/31.9
**averages →**	**33.1**	**30.4**	**30.4**	**31.6**

Table S5: Average results of analysis of populations of different elements of the secondary α-chymotrypsin structure in four mixing experiments: 20μM CHA with phosphate buffer (EXP A), 20μM CHA with 80 mM SDS (EXP B), mixture 20μM CHA/20mM SDS with 60 mM SDS (EXP C), and mixture 20μM CHA/6mM SDS with 74 mM SDS (EXP D), obtained from five programs indicated in the Table.

		secondary structure type					
EXP	program	H_α^R	H_α^D	S_β^R	S_β^D	T	U
A	SELCON3	6.0	4.7	18.5	10.8	19.8	36.6
	CDSSTR	2.6	5.2	20.3	12.0	23.6	35.0
	CONTIN	6.3	9.2	19.5	10.8	21.1	33.1
	BeStSel	11.4		24.0	7.2	14.0	43.4
B	SELCON3	14.0	15.2	10.6	7.4	22.9	30.6
	CDSSTR	17.3	16.3	9.8	7.3	21.1	28.3
	CONTIN	14.0	15.6	9.4	7.5	23.0	30.4
	BeStSel	26.9		12.0	3.5	14.5	43.0
C	SELCON3	14.0	15.2	10.7	7.4	23.0	30.5
	CDSSTR	17.2	16.3	9.4	7.3	21.3	28.6
	CONTIN	14.2	15.6	9.4	7.5	22.9	30.4
	BeStSel	26.4		12.1	3.1	14.4	44.1
D	SELCON3	11.2	12.7	13.6	8.4	22.3	31.0
	CDSSTR	12.8	13.3	12.6	8.6	21.9	30.6
	CONTIN	11.5	13.8	11.5	8.4	23.1	31.6
	BeStSel	21.1		16.0	2.8	14.8	45.4

References

(1) Micsonai, A.; Wien, F.; Kernya, L.; Lee, Y.-H.; Goto, Y.; Réfrégiers, M.; Kardos, J. Accurate Secondary Structure Prediction and Fold Recognition for Circular Dichroism Spectroscopy. *Proc. Natl. Acad. Sci. USA* **2015**, *11*, E3095–E3103.

(2) Micsonai, A.; Wien, F.; Bulyáki, E.; Kun, J.; Moussonog, E.; Lee, Y.-H.; Goto, Y.; Réfrégiers, M.; Kardos, J. BeStSel: A Web Server for Accurate Protein Secondary Structure Prediction and Fold Recognition from the Circular Dichroism Spectra. *Nucleic Acids. Res.* **2018**, *46*, W315–W322.

(3) Sreerama, N.; Woody, R. W. A self-consistent method for the analysis of protein secondary structure from circular dichroism. *Anal. Biochem.* **1993**, *209*, 32–44.

(4) Johnson, Jr., W. C. Analyzing protein circular dichroism spectra for accurate secondary structures. *Proteins: Struct. Funct. Genet.* **1999**, *35*, 307–312.

(5) Provencher, S. W.; Glöckner, J. Estimation of protein secondary structure from circular dichroism. *Biochemistry* **1981**, *20*, 33–37.

(6) Sreerama, N.; Woody, R. W. Protein secondary structure from circular dichroism spectroscopy. Combining variable selection principle and cluster analysis with neural network, ridge regression and self-consistent method. *J. Mol. Biol.* **1994**, *242*, 497–507.

(7) Sreerama, N.; Woody, R. W. Estimation of Protein Secondary Structure from Circular Dichroism Spectra: Comparison of CONTIN, SELCON, and CDSSTR Methods with an Expanded Reference Set. *Anal. Biochem.* **2000**, *287*, 252–260.
